# Prevalence and Sociodemographic Risk Factors of Soil-Transmitted Helminths in Rural Communities Living in Endemic Foci of Onchocerciasis in Southern Gabon

**DOI:** 10.3390/pathogens13110967

**Published:** 2024-11-06

**Authors:** Reinne Moutongo Mouandza, Jean Romain Mourou Mbina, Bridy Moutombi Ditombi, Joyce Coella Mihindou, Dimitri Ardrin Moussavou Mabicka, Christian Mayandza, Noe Patrick Mbondoukwe, Bedrich Pongui Ngondza, Luccheri Ndong Akomezoghe, Denise Patricia Mawili Mboumba, Marielle Karine Bouyou Akotet

**Affiliations:** 1 Department of Parasitology-Mycology and Tropical Medicine, Faculty of Medicine, Université des Sciences de la Santé, Libreville P.O. Box 4009, Gabon; reinneberthier76@yahoo.com (R.M.M.); mangondu20@yahoo.fr (J.R.M.M.); bridymoutombi@hotmail.com (B.M.D.); micojo93@yahoo.com (J.C.M.); dimitrimabicka7@gmail.com (D.A.M.M.); christianmayandza44@gmail.com (C.M.); mbondoukwenoe@yahoo.fr (N.P.M.); bedrich.pongui@gmail.com (B.P.N.); luccherindongakomezoghe@gmail.com (L.N.A.); dpmawili@gmail.com (D.P.M.M.); 2 Centre de Recherche en Pathogènes Infectieux et Pathologies Associées (CREIPA), Université des Sciences de la Santé, Libreville P.O. Box 4009, Gabon; 3 Centre de Formation et de Recherche en Microbiologie-Maladies Infectieuses et Tropicales (CREMMIT), Institute of Infectious Diseases Pr. Daniel Gahouma, Owendo P.O. Box 18141, Gabon

**Keywords:** soil-transmitted helminthiasis, onchocerciasis, co-endemicity, parasite prevalence, risk factors, Gabon

## Abstract

This prospective survey determined the prevalence and intensity of infections due to geohelminths and the associated risk factors in five onchocerciasis-endemic communities in Gabon between January and February 2020. Onchocerciasis endemicity was confirmed by Ov16 IgG testing. STHs were detected using the Kato–Katz techniques. Prevalence and parasite density were analyzed according to age, sex, and onchocerciasis endemicity. STHs were found in 64.8% of participants and were more common in hypoendemic (80.9%) and hyperendemic (63.9%) onchocerciasis communities (*p* < 0.01). *Ascaris lumbricoides* (65.5%), *Trichuris trichiura* (57.1%), and hookworms (17.3%) were prevalent in areas hypoendemic for onchocerciasis (*p *= 0.04). Male participants were more often infected by hookworms. Adults were more frequently infected by STHs (75.9%) than elders, who were the least infected (39.3%) (*p* < 0.01). Participants living in sporadic onchocerciasis areas frequently but not significantly harbored a moderate ascariasis load (6960.0 (1068.0–9492.0) eggs per gram) (*p *= 0.4). The highest parasite density was observed among participants aged 20–45 years (15,336.0 (3888.0–35,028.0)). A low *T. trichiuria* prevalence was found in all communities. STHs are highly prevalent in hypoendemic and hyperendemic areas and adults. An integrated combined approach of STH and onchocerciasis elimination through efficacious mass drug administration targeting all age groups should be considered.

## 1. Introduction

Neglected Tropical Diseases (NTDs) affect more than one billion individuals in the world [1]. Most NTDs share geographical distribution, common risk factors, and health or socio-economic impacts. Soil-transmitted helminth infections caused by intestinal worms such as *Ascaris lumbricoides *(Linnaeus, 1758),* Trichuris trichiura *(Linnaeus, 1771), hookworms (*Ancylostoma duodenale* (Dubini, 1843), and *Necator americanus *(Stiles,1902), and on the other hand, Onchocerciasis, also called river blindness, which is caused by *Onchocerca volvulus *(Leuckart, 1893), are parasitic NTDs that are highly endemic in sub-Saharan Africa. An estimated 1.5 billion people are infected by at least one STH species globally [2]. Furthermore, 99% of the 20.9 million onchocerciasis cases are in sub-Saharan Africa [3]. A recent report from national and community surveys highlighted the significant burden of STH in Gabon, with the highest rate (56.1%) recorded among school children [4,5,6,7,8]. The national prevalence of onchocerciasis in Gabon is estimated at 28.9%, with infection rates ranging from 0.1% to more than 80% in some villages. According to recent World Health Organization (WHO) estimates, 27 out of the 57 districts of the country require preventive chemotherapy for onchocerciasis [9]. In 2020, 76,000 out of 2,000,000 inhabitants needed effective treatment [10]. Moreover, polyparasitism, i.e., concurrent infection of an individual with two or more parasite species, can occur in areas with a high level of onchocerciasis [11].

Given the loiasis co-endemicity and the frequent *Loa loa* (Cobbold 1864) hypermicrofilaremia carriage in onchocerciasis foci; large-scale administration of ivermectin has not been performed in the country for more than 20 years [12]. Yet, new and safe mass drug administration (MDA) strategies for loiasis co-endemic areas for subjects with high *L. loa* microfilaremia are now possible [13,14]. Furthermore, several studies have demonstrated the effectiveness of preventive chemotherapy in reducing STH prevalence and morbidity after a decade [15,16]. Since ivermectin has been shown to be effective against geohelminths, especially when co-administered with albendazole for *T. trichiura* species, this treatment can also be an option in onchocerciasis foci with a high burden of STH [17,18]. African Programme for Onchocerciasis Control (APOC) projects were extended to hypoendemic areas, providing the opportunity to establish community-directed treatment with ivermectin (CDTI) in all endemic areas [19]. Thus, specific endemic settings of Gabon could be eligible for MDA and support the eradication of onchocerciasis. However, the level of STH–onchocerciasis co-endemicity must be determined in order to design effective chemoprevention strategies for onchocerciasis and other NTDs, including STH (i.e., ivermectin MDA or albendazole MDA with patient-centered curative treatment for onchocerciasis).

Integrating the local mapping and epidemiology of STH and onchocerciasis co-endemic areas would allow to address specific decision-making questions for the design of tailored effective control strategies in co-endemic areas at the national level. The purpose of this study was to estimate the prevalence and intensity of STH infections in remote communities in southern Gabon with different levels of onchocerciasis endemicity and to determine the sociodemographic factors associated with these infections.

## 2. Materials and Methods

### 2.1. Study Area and Population

#### 2.1.1. Study Sites

**Figure 1:**
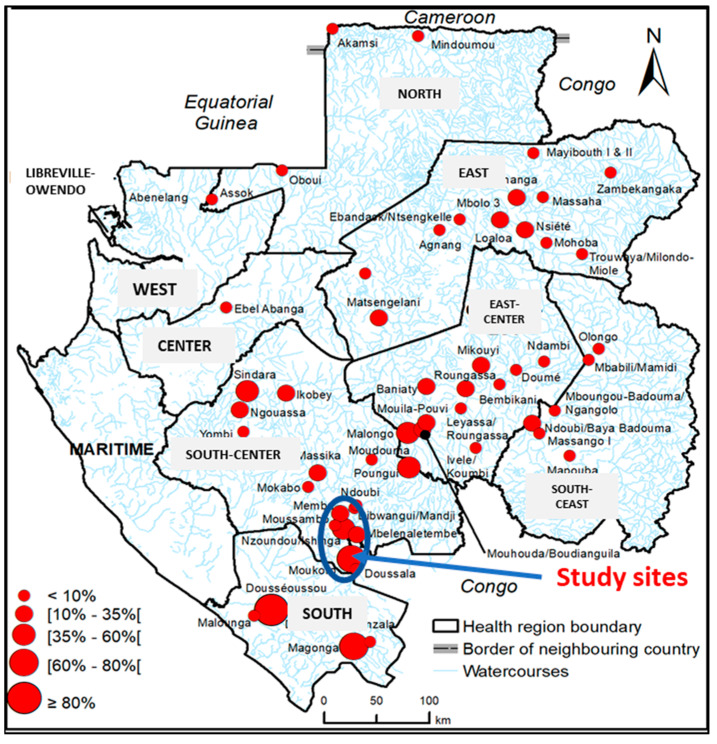
Study sites within onchocerciasis endemic settings [5].

This study was carried out in five historical onchocerciasis foci reported in the database of the National Control Programme of Parasitic Diseases (NCPPD) and the 2015 onchocerciasis endemicity estimates (Figure 1) [5]. These foci are located in the south of Gabon in the Louétsi-Wano department about 650 km from the capital city, Libreville. The five communities are in a rainforest environment and are 4 km to 40 km away from Lebamba, the main city in the department: Memba (4 km), Nzoundou-Issinga (10–14 km), Mayanga (20 km), Mbelnaletembe (28 km), and Matamatsengue-Nzingui (38–40 km). These localities include smaller healthcare facilities that provide primary care. All the participants in the studied communities are members of the same ethnic group (Nzebi) and share highly homogeneous habits and ways of life. Their main activities are agriculture, hunting, and fishing.

#### 2.1.2. Study Populations

All residents aged more than five years were invited to take part in this study. After explaining the purpose of this study, adult volunteers who gave their consent and children whose guardians gave their informed assent to participate were interviewed using a structured questionnaire. The sociodemographic information and medical history of all the participants were recorded.

#### 2.1.3. Sample Size Calculation

The required sample size for this study was determined as follows: n = Z^2^ × P (1 − P)/d^2^, with n = sample size, P = expected prevalence, d = precision (at 5% marginal error), and Z = standard score at a 95% confidence interval [20]. The prevalence of STH, estimated at 22.2% on the basis of data reported previously in the rural area in the south of Gabon, was used for calculation [21]. Therefore, the minimum sample size calculated for this study was 266.

### 2.2. Biological Testing

After an interview and physical examination, the included participants were given sterile and clean stool specimen containers for stool collection with the recommendation to immediately return it to the study team after collection. They were sampled for blood testing when they brought the collected stool back.

#### 2.2.1. Sample Collection

Ten microliters of whole blood were collected by a finger prick for the immediate detection of Ov16 antibodies in the field. Clear instructions for stool collection were given to each participant and parent. The participants were asked to fill half the container with stool. Stools were collected in the morning and immediately returned to the study team. However, some participants who did not provide the samples in the morning were allowed to bring back their stool later in the day. Depending on the proximity of the study site to the laboratory, stools were kept between 2 and 4 h at room temperature (22 to 25 °C).

Once in the laboratory, a quantity of 100 mg of stool, consisting of samples taken with a spatula from different sites of the stool provided by the participants, was used for the detection of parasites. The Kato–Katz technique was used for direct microscopy examination and quantification. Helminth culture was also performed to isolate and identify hookworms and *Strongyloides stercoralis* larvae.

#### 2.2.2. Kato–Katz Technique

The Kato–Katz technique was performed by experienced laboratory technicians according to Kato and Miura [22]. Briefly, an aqueous solution of 3% of green malachite was mixed with 100 mL of glycerol and 100 mL of distilled water. Cellophane coverslips were previously soaked in this solution and then pressed on a thick smear of 41.7 mg of fecal sample. The slides were read between half an hour and one hour after preparation to avoid the destruction of hookworm eggs. The slides were kept longer for other intestinal helminths. *A. lumbricoides* and *T. trichiura* parasite densities were calculated according to the Kato–Katz protocol and expressed as the number of eggs per gram of feces. The intensity of STH was determined in eggs per gram of feces (epg) in positive samples as follows: *A. lumbricoides* (low: 1–4999 epg; moderate: 5000–49,999 epg; high: ≥50,000 epg) and *T. trichiura* (low: 1–999 epg; moderate: 1000–9999 epg; high: ≥10,000 epg).

#### 2.2.3. Helminth Culture

STH culture was performed according to the Harada and Mori method previously described [23]. Briefly, about 1 g of stool sample was spread on a slide and covered with filter paper in the presence of sterile water in a Petri dish. The preparation was incubated for seven days at 25 °C. The water was collected and centrifuged, and the sediment was examined under a microscope on days three, five, and seven. This allowed for the detection of hookworm and *Strongyloides stercoralis* (Bavay, 1876) larvae. Differential diagnosis between these species was performed based on morphology after seven days of culture [24]. Parasite culture was considered positive if at least one larva was present in the sample. Participants with more than one parasite species identified were considered coinfected. Polyparasitism was defined in the case of two or more parasite species in a given stool sample.

#### 2.2.4. Onchocerciasis Seropositivity

The rapid diagnostic test (RDT) Ov16 of SD Bioline (Abbot standard diagnostics, Inc. Yongin, Republic of Korea) was used to estimate the prevalence of Immunoglobulin IgG4 Ov 16 of *O. volvulus* in the five villages and to assess the ongoing local transmission of onchocerciasis [25]. The qualitative detection of IgG4 antibodies against Ov16 was performed by trained technicians according to the manufacturer’s recommendations. The results were available after 20 min and were read separately by two different technicians.

Based on previous data which showed a high concordance between skin snip and the prevalence of Ov16 antibodies (IgG4) in a population never treated with ivermectin and considering the lack of MDA or in another intervention in these communities, onchocerciasis endemicity groups were defined according to RDT interpretation [5,26]. Onchocerciasis endemicity was defined as (i) sporadic, for villages where the positivity rate of RDT was below 10%; (ii) hypoendemic, when the positivity rate ranged between 10 and 35%; and (iii) hyperendemic, when it was higher than 60%.

### 2.3. Statistical Analysis

Data were recorded in an Excel spreadsheet and analyzed using Statview 5.0 software (SAS Institute, Cary, NC, USA). The normality of collected data was checked with the Shapiro test and the Kolmogorov–Smirnov test for quantitative variables. Since none of the variables followed a normal distribution, quantitative variables were presented in medians with interquartile ranges, and comparisons between groups were performed using the Mann–Whitney and the Kruskal–Wallis tests. Frequencies were compared using a chi-squared test or Fischer’s exact test. For the logistic regression analysis, the outcome variable was the presence of any STH carriage. Independent variables were onchocerciasis endemicity and participant age. Participants were classified according to age as follows: <11 years (children), 11–18 years (adolescents), 19–59 years (adults), and ≥60 years (elders). Associations of onchocerciasis endemicity and age categories with the presence of ascariasis and/or trichiniasis were assessed using different multinomial regression models. In the first model, the type of endemicity, age group, sex, educational level, and previous anthelminthic treatment were considered as confound factors. When groups according to the type of STH infections were predefined, the model only considered the factors that were found associated with the presence of at least one STH with a *p* < 0.2 in the bivariate analysis as confound factors. The adjusted odds ratio (aOR) for the logistic regression results and the crude odds ratio (cOR) for the bivariate analysis results were reported at a 95% confidence interval (95% CI). All *p*-values were two-tailed, and *p* < 0.05 was considered statistically significant.

## 3. Results

### 3.1. General Characteristics of the Study Population

Among the 513 inhabitants of the five communities, 159 of them were not included: 65 children were aged under five years, 22 did not give back the stool container and refused to be sampled for Ov16 IgG4 detection, 21 did not accept to participate in this study, and 46 were absent during the study period. Therefore, a total of 359 volunteers were enrolled and had blood and stool testing. Their median age was 22 (10–48) years, and adults and elders represented 57.2% of participants. Only 10% of volunteers reported using a self-treatment with an anthelminthic in the past 3 months before this study. The majority of participants (80.5%, n = 299) had a primary or secondary school level (Table 1). Only five participants had semi-brick-wood dwellings; the others had wood dwellings. Participants used non-conventional latrines, they consumed river or stream water whether or not associated with tap water. More than two-thirds of the participants did not regularly wash their hands (Table 1).

### 3.2. Prevalence of Ov16 IgG Seropositivity and Onchocerciasis Endemicity

The global prevalence of *O. volvulus* IgG4 seropositivity was 24.2% (95% CI 20.0–30.0). The highest positivity rate was found at Nzoundou-Issinga with 69.4% (95% CI 57.1–79.4; n = 43/62), and it was defined as an onchocerciasis hyperendemic area. Memba had the lowest positivity rate, less than 5% (95% CI 1.9–11.9; n = 4/82), and as such, it was defined as an onchocerciasis sporadic area. The positivity rate of the other three communities, Mayanga (21.9%, 95% CI 11.0–38.8; n = 7/32), Mbelnaletembe (14.2%, 95% CI 7.4–25.7; n = 8/56), and Matamatsengue-Nzingui (20.0%, IC 95% 13.9–27.9, n = 25/127), ranged between 10 and 29.3%, and these were defined as onchocerciasis hypoendemic areas.

### 3.3. STH Prevalence

Overall, 298 participants provided stool samples. Age was not recorded for four of them. Among the 298 samples examined, 193 (64.8%) carried at least one STH. Polyparasitism was detected in 55.4% of infected participants (n = 107/193), while monoparasitism was found in 44.6% (n = 86/193) (Table 2). The prevalence of STH varied significantly according to communities. The prevalence was 26.1% (n = 18/69) in the onchocerciasis sporadic area, 80.9% (n = 136/168) in the onchocerciasis hypoendemic localities, and 63.9% (n = 39/61) in the onchocerciasis hyperendemic area (*p* < 0.01) (Table 2).

### 3.4. Prevalence and Intensity of Intestinal Nematode Species

*Ascaris lumbricoides* was the most prevalent species (48.3%, n = 144/298), followed by *T. trichiura* (43.9%, n = 131/298), hookworms (11.4%, n = 34/298), and *S. stercoralis* (2.0%, n = 6/298). No *Schistosoma *species was detected (Table 3). *A. lumbricoides* and *T. trichiura* infections were commonly co-detected (25.2%, n = 75/298). Single infections with *A. lumbricoides* were found in 14.8% of cases of mono-infection (n = 44/298), followed by *T. trichiura* single infections (11.4%; n = 34/298) (Table 2).

Only six (2.0%) participants had a single hookworm infection. Hookworms were frequently associated with ascariasis or trichiniasis (10.3%, n = 11/107, respectively) (Table 2). Infected participants living in the onchocerciasis sporadic area mainly carried one parasite species (88.9%; n = 16/18). Polyparasitism was more common in communities living in onchocerciasis hypoendemic settings (44.6%) compared to other sites (53.0%, n = 89/168) (*p* < 0.01) (Table 2).

Only six (7.0%) participants had a single hookworm infection. Hookworms were frequently associated with trichiniasis or ascariasis (10.3%, n = 11/107, respectively) (Table 2). Infected participants living in the onchocerciasis sporadic area often carried one parasite species (23.2%; n = 16/69). Polyparasitism was more common in communities living in onchocerciasis hypoendemic settings (53.0%, n = 89/168) compared to other sites (2.9% for the onchocerciasis sporadic area and 26.2% for the onchocerciasis hyperendemic area) (*p* < 0.01) (Table 2).

**Figure 2:**
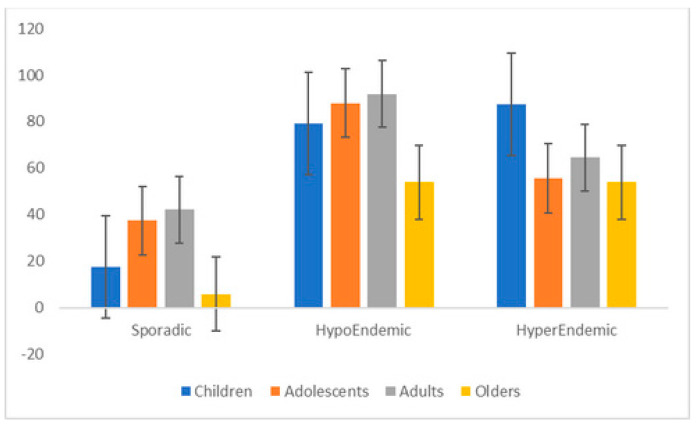
Prevalence (with 95% CI) of STH carriers according to age and onchocerciasis endemicity.

While the prevalence of STH carriers was high in the onchocerciasis hypoendemic areas, it was significantly low in the onchocerciasis sporadic area (Figure 2). The 14 persons who carried three different parasite species lived in the onchocerciasis hypoendemic site (Table 2). The proportion of women with at least one STH (63.2%, n = 96/152) was comparable with that of males (66.4%, n = 97/146) (*p* = 0.63). According to the age, STH prevalence was 64.7% (n = 44/68), 69.0 (n = 40/58), 75.9% (n = 85/112), and 39.3% (n = 22/56) in children, teenagers, adults, and elders, respectively (*p* < 0.01) (Figure 2). The distribution according to age differed significantly between sites. The rate of STH carriage was the lowest in all age groups in the onchocerciasis sporadic community, mainly in elders (5.9%; 95% CI 0.1–28.7) compared to children (17.6%; 95% CI 3.8–51.6) (*p* = 0.03). The majority of adults (91.9%; 95% CI 82.2–97.3) and adolescents (87.9%; 95% CI 71.8–96.6) carried intestinal parasites in the hypoendemic community. The prevalence of STH was comparable among elders living in the onchocerciasis hypoendemic community (53.8%; 95% CI 33.4–73.4) and hyperendemic community (53.8%; 95% CI 25.1–80.8) (Figure 2).

Egg density per gram (epg) was recorded for *A. lumbricoides and T. trichiura*. The *A. lumbricoides *parasite load ranged from low (24 epg) to high (41,592 epg), with a median of 3792 epg (90–9924). *Trichuris trichiura* epg remained below 10,000 epg, with a median of 228 (120–816) epg.

The *A. lumbricoides* parasite load ranged from low (24 epg) to high (41,592 epg), with a median of 3828 (696–10,248) epg. *T. trichiura* epg remained below 10,000 epg, with a median of 228 (120–616) epg.

### 3.5. STH Species Prevalence and Parasite Density According to Onchocerciasis Endemicity

*Ascaris lumbricoides, T. trichiura,* and hookworm infection rates were significantly low (11.6%, 11.6%, and 5.8%, respectively) in the onchocerciasis sporadic community (*p* < 0.01). However, infection rates were the highest in onchocerciasis hypoendemic areas (65.5%, 57.1%, and 17.3%, respectively). Only one participant in the onchocerciasis hyperendemic village was infected with hookworms (Table 3).

**Figure 3:**
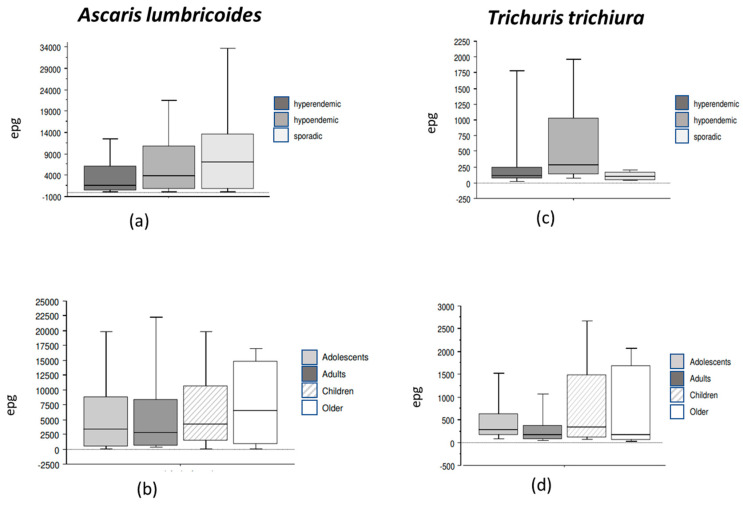
Median intensity of helminth infection according to onchocerciasis endemicity and age. The intensity *of A. lumbricoides* infection according to onchocerciasis endemicity (**a**) and age (**b**); the intensity of *T. trichiura* infection according to onchocerciasis endemicity (**c**) and age (**d**). Bars represent the 25th and the 75th percentiles.

*Ascaris lumbricoides* parasite density tended to be low when onchocerciasis endemicity was high (onchocerciasis hyperendemic area: 1704 (432–6156); onchocerciasis hypoendemic area: 4032 (888–11,496); onchocerciasis sporadic area: 7128 (792–13,620) (Figure 3a) (*p* = 0.08). However, participants living in the three onchocerciasis hypoendemic communities had the highest *T. trichiura* egg density (Figure 3b).

### 3.6. Intestinal Nematode Prevalence and Intensity According to Age and Gender

All STH infection rates and intensities were comparable between males and females: 3504 (660–8712) epg vs. 4572 (816–11,496) epg for *A. lumbricoides* and 216 (120–648) vs. 240 (96–888) epg (*p* = 0.76) for *T. trichiura*.

Although *A. lumbricoides* median egg density did not significantly vary between age groups (*p* = 0.73), children (4200 (1452–10,662) epg) and elders (6528 (966–14,880) epg) had the highest parasite densities (Figure 3c). *Trichuris trichiura* parasite density tended to be elevated with age, it was the highest in children (336 (126–1482) epg) and the lowest in adults (168 (90–378) epg) and elders (180 (72–1680) epg), in the group of infected adolescent *Trichuris trichiuraImedian* density was 288 (174–636) epg) (*p* = 0.06) (Figure 3d).

According to gender, the prevalence of *A. lumbricoides* was high in females (52.0%, n = 79/152), while that of *T. trichiura* was high in males (44.5%, n = 70/146), without a statistical difference (*p* = 0.19 and *p* = 0.17, respectively) (Table 4). Hookworm infection rates were high in males (15.9%, n = 22/145) (*p* = 0.05). *Strongyloides stercoralis* larvae were found in males only (Table 3).

According to group age*, A. lumbricoides* and *T. trichiura* were most frequent in adults (54.5%, n = 61/112 and 53.6%, n = 60/112, respectively) compared to other age groups. No statistical difference was found between age groups for hookworm carriage (*p* = 0.13)*. Strongyloides stercoralis* larvae were not detected in children (Table 3).

### 3.7. STH Species Prevalence and Intensity According to Previous Antihelminthic Treatment

*Ascaris lumbricoides* and *T. trichiura* infection rates were low among participants who reported previous antihelminthic self-treatment. No hookworm infection was found in this group (Table 3). Although the median density of eggs of *A. lumbricoides* tended to be two-fold higher in participants who did not take antihelminthic treatments (3864 (696–10,326) epg) compared to those who had (1800 (743–7554) epg), the difference was not statistically significant (*p* = 0.541). Comparable egg densities were observed between participants who took drugs (204 (120–540) epg) and those who did not (228 (120–840) epg) (*p* = 0.98).

### 3.8. STH Species Prevalence and Intensity According to Level of School Attendance

Two-thirds (67.8%, n = 135/199) of infected participants had a primary school level. The same trend was observed for all STH species (Table 3). Participants with a secondary school degree were less frequently infected by all STH species and had no *S. stercoralis* larvae (Table 3). *Ascaris lumbricoides* median egg density was 6228 (1500–16,284) epg among participants with no school attendance, 3528 (726–9810) epg in those with primary school level, and 1872 (438–7782) epg in the group of participants with a secondary school level (*p* = 0.04). However, *T. trichiura* median egg densities were similar in the absence of school attendance (240 (138–1032) epg or primary school level (240 (120–864) epg) (*p* = 0.2).

### 3.9. Factors Associated with STH Carriage

After multivariate analysis, living in a hypoendemic or hyperendemic onchocerciasis area was a risk factor for STH carriage (Table 4). Other associated factors were age between 11 and 60 years old (*p* = 0.03) mainly for *A. lumbricoides*. The absence of previous treatment with an antihelminthic was also identified as a risk factor. The group of elders had the lowest risk *of T. trichiura* carriage (Table 4).

Irregular wearing of shoes outside dwellings was more common in onchocerciasis hypoendemic villages (32.0%) compared to sporadic (12.6%) and hyperendemic (5.3%) ones (*p* < 0.01). However, it was not associated with STH carriage. Indeed, it was reported by 46.9% of the STH carriers and by 48.1% of the uninfected ones (*p = *0.51). The six participants who drank exclusively tap water lived in the sporadic area.

## 4. Discussion

The Gabonese government is committed to the integrated control of national public health problems in the country in a challenging context of limited resources and remote areas. Thus, it is essential to identify areas where tailored disease control strategies would be designed. With the shift from onchocerciasis control to elimination, all endemic areas would become eligible for MDA using ivermectin. This may also affect STH, particularly *A. lumbricoides*, and to a lesser extent hookworm and whipworm species. This study, which is the first integrated assessment of the prevalence of STH and onchocerciasis in co-endemic villages of Gabon, was performed to provide critical information for the control and integrated elimination of these NTDs that are targeted for global elimination by 2030 [27]. The elimination of both types of NTDs requires surveys to determine eligibility, continuity, or cessation of mass interventions, including MDA. This strategy is part of the 2021–26 national strategic plan for the public health of Gabon.

Previous data classified Gabon as a hypoendemic country for onchocerciasis [28]. However, different levels of onchocerciasis endemicity exist across the country, as described in a survey conducted by the NPDCP and a recent meta-analysis [5,29]. Positive skin snip prevalence rates recorded by the NPDCP five years ago in the five study sites were 97% for Ishinga, 22% for Mbelnaletembe and Nzingui, 17.2% for Mayanga, and 10% for Memba [30]. Our study highlights the continuous circulation of *O. volvulus* in these historical foci. Indeed, the overall prevalence of onchocerciasis was 24.2%. Data on the evolution of filariasis prevalence exist for areas where MDA with ivermectin has been performed, while such information is lacking in areas without any control intervention. However, in areas of Congo and Cameroon where the prevalence of another filariasis, loiasis, was compared over a 7- to 10-year period without specific interventions, such as screening and treatment of cases, no significant changes were observed [31]. The absence of modification of the ecological environment which is favorable to the development of sand flies, the absence of specific health intervention for more than 30 years in the study sites, and the infrequent migration of the population (mainly adults and older members of the communities who are the most exposed) of these very remote areas are factors which may explain the observed same level of onchocerciasis prevalence throughout the years. Thus, these historical foci could be eligible for MDA. Indeed, the *L. loa* hypermicrofilaremia rate is very low in these sites [32]. Considering the high prevalence rate, which reached 64.8%, STHs remain a major public health problem in Gabonese remote rural communities. A similar rate was observed among rural communities in southwestern Ethiopia (70.5%) [33]. This high prevalence could also be explained by the permanent exposure of inhabitants to unsanitary water and low socio-economic conditions. On the other hand, polyparasitism was also frequent (35.9%) (Table 2). Thus, there is an urgent need to pursue the mapping of STH across the country in order to implement appropriate control strategies. The studied onchocerciasis areas are also endemic for STH. The coexistence of these NTDs is not surprising in our tropical climate.

Although skin snips remain the gold standard for onchocerciasis diagnosis, it is labor intensive, it needs well-trained technicians, and it is not well accepted by communities [34]. Serological detection of IgG4 Ov16 is used in decision making for stopping MDA and for parasite circulation detection [35]. SDbioline RDT was shown to have higher sensitivity for *O. volvulus* diagnosis compared to skin snip and a higher specificity compared to Elisa [26]. According to onchocerciasis endemicity determined by Ov16 seropositivity rates, hypoendemic areas had the highest STH prevalence (80.9%), followed by hyperendemic areas (63.9%), while the lowest prevalence rate (26.1%) was found in the sporadic onchocerciasis community (Table 2). This discrepancy could be due to the difference in exposure to the parasites and the intensity of the environmental reservoir. In fact, the low prevalence rate observed in the sporadic area may be explained by better access to healthcare and the more frequent use of safe water through public hydraulic pumps, given that this community lives near the main city (only 4 km away). The other onchocerciasis communities are far from Lébamba, the main city, and have impracticable roads, especially during the rainy season. In these communities, the river was the main source of drinking water, despite the known increased risk of STH associated with river water [36]. These results also reflect the precarious living conditions of these populations and, most probably, favorable conditions for the development of *Simulium *sp. As such, these communities have permanent exposure to both types of parasitic infections.

In regions co-endemic or sharing factors of exposure to various pathogens, it is recommended that prevalence or impact assessments should be integrated [37,38]. The present integrated survey will enable the health authorities to decide, in an integrated way, on control strategies of STH and onchocerciasis in Gabon. As an example, the integrated assessment of the prevalence of onchocerciasis and lymphatic filariasis (LF) in three co-endemic regions of Ethiopia has made it possible to provide important data on the endemicity of onchocerciasis in these regions. Two programmatic decisions were taken: to stop MDA for LF and to continue MDA for onchocerciasis [31].

Concerning polyparasitism, the number of nematode species per individual was high when the prevalence of STH was also high (Table 2). Polyparasitism was found in more than half of the study population. *Ascaris lumbricoides-T. trichiura* co-infection was commonly encountered (25.2%). This finding is in line with reports from southern Ethiopia [39]. Single and multiple parasitic infections were observed in the onchocerciasis hyperendemic and hypoendemic settings, while polyparasitism was rare in the onchocerciasis sporadic setting, where, surprisingly, the STH median density was the highest (Figure 3a). No conclusions can be drawn from these results. Nevertheless, it is known that interactions between parasites may be antagonistic or synergistic [40,41].

STH prevalence increased with age. Adolescents and adults had the highest STH prevalence (69.0% and 75.9%) (Figure 2, Table 3). Inversely, *A. lumbricoides* median egg density tended to be the lowest in adolescents, while the highest parasite load was observed in the elders group (Figure 3b). These data are not consistent with those from Cameroon, where the prevalence rate and intensity of *A. lumbricoides* parasite decreased as age increased [42]. The poor use of STH preventive measures and the outdoor activities of adults who are also not targeted by chemoprevention campaigns may explain these findings [33]. Thus, there is a strong need to include adults in the periodic mass deworming programs to control and eliminate STH transmission, as recommended by the WHO. Indeed, untreated adults remain reservoirs of parasites and cause household reinfections by STH, maintaining parasite transmission in the community [43]. The apparent association between *O. volvulus* endemicity and these STH prevalence rates could partly be explained by the fact that communities live in areas where both disease exposure and risk factors are present. Indeed, hyperendemic sites are those located in very remote settings, with no access to safe water and insufficient sanitary conditions. It is obvious that the study populations are exposed to both types of parasitic infections. A study conducted in children in 2005 reported that *O. volvulus*-infected pupils were more likely to be infected with *A. lumbricoides* than *O. volvulus*-negative pupils [44]. Additional data are needed to confirm this relationship. MDA with ivermectin to control onchocerciasis should be considered in these communities. This broad-spectrum anthelminthic is also used to treat STH [45]. Furthermore, the co-administration of ivermectin with albendazole or mebendazole would be an optional treatment to improve therapeutic outcomes [46].

High *T. trichiura* parasite densities were found in the onchocerciasis hypoendemic areas. These results would confirm the co-incidence of STH and onchocerciasis in the study areas, with a probable different level of *O. volvulus* vector circulation. The community members are exposed to STH in all five villages, while *O. volvulus* endemicity differs between them.

An integrated approach to controlling NTDs with the same geographical distribution using molecules for treatment or prevention, as well as the distribution of drugs active against several pathogens, is currently advocated by the NTD RoadMap [10]. The benefits of ivermectin MDA in reducing the prevalence of STHs have been demonstrated in several co-endemic regions [47]. Ivermectin has therapeutic efficacy against *S. stercoralis, A. lumbricoides, and T. trichiura*, the main STH found in Gabon [6,7,48,49]. Actually, the success of preventive chemotherapy is threatened by resistance to anthelmintics and the poor efficacy of drugs against hookworms (mebendazole) and *T. trichiura* (albendazole and mebendazole). Ivermectin has been shown to be effective against geohelminths in several studies, mainly on ascariasis. In areas such as Gabon where loiasis, onchocerciasis, and STH are co-endemic, using ivermectin co-administered with albendazole or mebendazole could be a good option.

This study has some limitations. The small number of STH-infected individuals in some groups does not allow us to draw strong conclusions regarding some associations. The prevalence rates of STH could be underestimated by the use of microscopic techniques. Parasites were detected by common microscopic tests which are usually used during surveys and for control strategy decisions. However, submicroscopic parasite carriers could have been misclassified as uninfected participants. Moreover, the onchocerciasis concomitant microfilaria density was not performed as well as ELISA, which could lead to an underestimation of the parasite burden.

In addition, the prevalence rates of STH could also be underestimated because the participants only provided one stool sample.

Our results should be confirmed with larger studies which will include the analysis of several stool samples per individual and the molecular detection of STH. Nevertheless, the Ov16 RDT was useful for rapid confirmation of onchocerciasis transmission intensity in areas where the filariasis circulation is known and where no control intervention was performed for more than 20 years.

## 5. Conclusions

STH prevalence increases with the level of onchocerciasis endemicity in the historic foci of onchocerciasis in Gabon. The age distribution of STH carriers varies according to onchocerciasis endemicity. The interaction between *O. volvulus* and STH should be investigated to understand the discrepancies observed in prevalence rates and densities. Further investigations on NTD co-endemicity prevalence and features in all age groups, as well as risk factors, should lead to the implementation of effective preventive chemotherapy measures. Therefore, such community-based STH data, including those reported in this study, would be important for prioritizing onchocerciasis endemic areas for MDA using ivermectin and potentially ivermectin plus albendazole to increase efficacy against hookworms and whipworm.

## Figures and Tables

**Table 1:** Study sites and population characteristics.

	N	%
Communities		
Memba	82	22.8
Mayanga	32	8.9
Mbelnaletembe	56	15.6
Nzingui/Matamatsengue	127	35.4
Issinga/Nzoundou	62	17.3
Age range *		
Children	68	23.1
Adolescents	58	19.7
Adults	112	38.1
Elders	56	19.1
Gender		
Male	179	49.9
Female	180	50.1
School attendance		
None/pre-school	70	19.5
Primary school	244	68.0
Secondary school	45	12.5
Previous treatment with an antihelmintic		
Yes	36	10.0
No	323	90.0
Wearing shoes when outside		
Sometimes	179	49.9
Regularly	180	50.1
Source of drinking water		
River and rain	282	78.6
River and/or tap	77	21.4
Hand washing after defecation		
Always	80	22.3
Sometimes	251	69.9
Never	28	7.8

* Age unknown for four participants.

**Table 2:** Soil-transmitted helminth distribution in the overall population according to onchocerciasis endemicity level.

			Overall Population		Onchocerciasis Endemicity						*p*-Value
					Sporadic		Hypoendemic		Hyperendemic		
			N	%	n	%	n	%	n	%	
N			298	-	69		168		61	-	
Positive			193	64.8	18	26.1	136	80.9	39	63.9	<0.01
	Monoparasitism		86	28.8	16	23.2	47	30.0	23	37.7	<0.01
		* A. lumbricoides *	44	14.8	6	8.7	28	16.7	10	16.4	
		* T. trichiura *	34	11.4	6	8.7	17	10.1	11	18.0	
		Hookworms	6	2.0	3	4.3	2	1.2	1	1.6	
		* S. stercoralis *	2	0.6	1	1.4	0	0.0	1	1.6	
	Polyparasitism		107	35.9	2	2.9	89	53.0	16	26.2	0.01
		*A. lumbricoides*—*T. trichiura*	75	25.2	2	2.9	57	33.9	16	26.2	
		*T. trichiura*—hookworms	7	2.3	0	0.0	7	4.2	0	0.0	
		*A. lumbricoides*—hookworms	11	3.7	0	0.0	11	6.5	0	0.0	
		*A. lumbricoides*—*T. trichiura* Hookworms	11	3.7	0	0.0	11	6.5	0	0.0	
		*A. lumbricoides*—*T. trichiura* —*S. stercoralis*	3	1.0	0	0.0	3	1.8	0	0.0	

**Table 3:** Prevalence of STH infection according to the study population characteristics.

Characteristic	*A. lumbricoides*				*T. trichiura*				Hookworms				*S. stercoralis*			
	n	%	95% CI	*p*-Value	n	%	95% CI	*p*-Value	n	%	95% CI	*p*-Value	n	%	95% CI	*p*-Value
Onchoendemicity				<0.01				<0.01				<0.01				0.835
Sporadic	8	11.6	5.1–21.6		8	11.6	5.1–21.6		4	5.8	1.6–14.2		2	2.9	0.3–10.1	
Hypoendemic	110	65.5	57.8–72.6		96	57.1	49.3–64.7		29	17.3	11.9–23.8		3	1.8	0.4–5.1	
Hyperendemic	26	42.6	30.0–55.9		27	44.3	31.5–57.5		1	1.6	0.0–8.8		1	1.6	0.0–8.8	
Gender				0.19				0.17				0.05				0.01
Female	79	52.0	43.7–60.1		61	40.1	32.3–48.4		12	7.9	4.1–13.4		0	0.0	0.0–24.0	
Male	65	44.5	36.3–53.0		70	47.9	39.6–56.4		22	15.1	9.7–21.9		6	4.1	1.5–8.7	
Age groups *				0.03				<0.01				0.13				0.01
Children	31	45.6	33.4–58.1		33	48.5	36.2–61.0		5	7.3	2.4–16.3		0	0.0	0.0–5.2	
Adolescents	32	55.2	41.5–68.2		25	43.1	30.2–56.8		10	17.2	8.6–29.4		2	3.4	0.4–11.9	
Adults	61	54.5	44.8–63.9		60	53.6	43.9–63.0		15	13.4	7.7–21.1		2	1.8	0.2–6.3	
Elders	18	32.1	20.3–46.0		12	21.4	11.6–34.4		3	5.3	1.1–14.9		1	1.8	0.0–9.5	
History of anthelminthic treatment in the last 3 months																
				<0.01				0.72				0.02				0.70
Yes	9	26.5	12.9–44.4		14	41.2	24.6–59.3		0	0.0	0.0–10.3		1	2.9	0.0–15.3	
No	135	51.1	44.5–57.3		117	44.3	38.2–50.5		34	12.9	9.1–17.5		5	1.9	0.6–4.4	
School attendance																
				0.53				0.64				0.23				0.54
None/pre-school	29	46.8	44.0–59.9		24	38.7	26.6–51.9		5	8.1	2.7–17.8		2	3.2	0.4–11.2	
Primary school	100	51.8	44.5–59.0		90	46.6	39.4–53.9		27	14.0	9.4–19.7		4	2.1	0.5–5.2	
Secondary school	15	40.5	24.7–57.9		17	45.9	29.5–63.1		2	5.4	0.7–18.2		0	0.0	0.0–9.4	

* Age unknown for two participants.

**Table 4:** Multivariate analysis of factors associated with ascariasis and trichiniasis.

	All STH					Ascariasis			Trichuriasis		
	N	%	aOR	95% CI	*p*-Value	aOR	95% CI	*p*-Value	aOR	95% CI	*p*-Value
Onchocerciasis endemicity					<0.01			<0.01			<0.01
Sporadic	18	26.1	1			1			1		
Hypoendemic	136	80.9	14.5	6.8–34.6		17.4	7.9–43.1		9.9	4.7–23.6	
Hyperendemic	39	63.9	5.6	2.4–14.6		7.2	2.9–19.6		6.1	2.6–15.6	
Gender					0.20			0.08			0.14
Female	96	63.1	1			1			1		
Male	97	66.4	0.8	0.4–1.4		0.61	0.4–1.1		1.4	0.9–2.2	
Age groups *					0.03			0.06			<0.01
Children	44	64.7	1			1			1		
Adolescent	40	69.0	1.1	0.7–3.0		1.8	0.8–4.2		0.8	0.6–1.5	
Adults	85	75.9	1.4	0.8–2.6		1.4	0.7–2.9		1.2	0.4–1.6	
Elders	22	39.3	0.6	0.3–1.2		0.6	0.3–1.4		0.3	0.1–0.6	
History of anthelminthic treatment in the last 3 months					<0.01			<0.01			0.76
No	177	67.0	1			1			1		
Yes	16	47.0	0.3	0.1–0.7		0.3	0.1–0.6		0.9	0.4–1.8	
Educational level					0.53			0.6			0.08
None/pre-school	36	58.1	1			1			1		
Primary school	135	70.0	1.2	0.6–2.0		1.1	0.5–2.3		1.3	0.7–2.3	
Secondary school	22	59.4	0.8	0.3–1.8		1.7	0.6–4.8		1.3	0.6–3.1	

* Age unknown for two participants.

## Data Availability

All relevant data are within the manuscript. Data and materials are available upon request from the corresponding author for any further information.

## References

[1] World Health Organization Global Report on NTD 2024. https://www.who.int/teams/control-of-neglected-tropical-diseases/global-report-on-neglected-tropical-diseases-2024.

[2] World Health Organization (2012). Soil-Transmitted Helminthiases: Eliminating as Public Health Problem Soil-Transmitted Helminthiases in Children: Progress Report 2001–2010 and Strategic Plan 2011–2020.

[3] Moser W., Schindler C., Keiser J. (2017). Efficacy of recommended drugs against soil transmitted helminths: Systematic review and network meta-analysis. BMJ.

[4] Sumbele I.U.N., Otia O.V., Bopda O.S.M., Ebai C.B., Kimbi H.K., Nkuo-Akenji T. (2021). Polyparasitism with Schistosoma haematobium, Plasmodium and soil-transmitted helminths in school-aged children in Muyuka-Cameroon following implementation of control measures: A cross sectional study. Infect. Dis. Poverty.

[5] World Health Organization (2015). Rapport de mission de cartographie de l’onchocercose et de la loase dans 69 village au Gabon. African Programme for Onchocerciasis Control (APOC).

[6] M’Bondoukwe N.P., Kendjo E., Mawili-Mboumba D.P., Koumba Lengongo J.V., Offouga Mbouoronde C., Nkoghe D., Toure F., Bouyou-Akotet M.K. (2018). Prevalence of and risk factors for malaria, filariasis, and intestinal parasites as single infections or co-infections in different settlements of Gabon, Central Africa. Infect. Dis. Poverty.

[7] Dejon-Agobe J.C., Honkpehedji Y.J., Zinsou J.F., Edoa J.R., Adegbite B.R., Mangaboula A., Agnandji S.T., Mombo-Ngoma G., Ramharter M., Kremsner P.G. (2020). Epidemiology of Schistosomiasis and Soil-Transmitted Helminth Coinfections among Schoolchildren Living in Lambarene, Gabon. Am. J. Trop. Med. Hyg..

[8] Zouré H.G., Noma M., Tekle A.H., Amazigo U.V., Diggle P.J., Giorgi E., Remme J.H. (2014). The geographic distribution of onchocerciasis in the 20 participating countries of the African Programme for Onchocerciasis Control: (2) pre-control endemicity levels and estimated number infected. Parasites Vectors.

[9] World Health Organization (2022). Expanded special Project for Elimination of NTD. Gabon 2020: Status of Onchocerciasis Elimination.

[10] World Health Organization (2020). Ending the Neglect to Attain the Sustainable Development Goals: A Road Map for Neglected Tropical Diseases 2021–2030.

[11] Steinmann P., Utzinger J., Du Z.-W., Zhou X.-N. (2010). Multiparasitism a neglected reality on global, regional and local scale. Adv. Parasitol..

[12] Kombila M., Richard-Lenoble D. (1998). The Mectizan donation program in Gabon: Progress and perspectives of distribution in the focus of onchocerciasis (1991–1997). Sante.

[13] Kamgno J., Pion S.D., Chesnais C.B., Bakalar M.H., D’Ambrosio M.V., Mackenzie C.D., Nana-Djeunga H.C., Gounoue-Kamkumo R., Njitchouang G.-R., Nwane P. (2017). A Test-and-Not-Treat Strategy for Onchocerciasis in Loa loa–Endemic Areas. N. Engl. J. Med..

[14] Blok D.J., Kamgno J., Pion S.D., Nana-Djeunga H.C., Niamsi-Emalio Y., Chesnais C.B., Mackenzie C.D., Klion A.D., Fletcher D.A., Nutman T.B. (2021). Feasibility of Onchocerciasis Elimination Using a “Test-and-not-treat” Strategy in Loa loa Co-endemic Areas. Clin. Infect. Dis. Off. Publ. Infect. Dis. Soc. Am..

[15] Bah Y.M., Bah M.S., Paye J., Conteh A., Saffa S., Tia A., Sonnie M., Veinoglou A., Amon J.J., Hodges M.H. (2019). Soil-transmitted helminth infection in school age children in Sierra Leone after a decade of preventive chemotherapy interventions. Infect. Dis. Poverty.

[16] Tabi E.S.B., Eyong E.M., Akum E.A., Love J., Cumber S.N. (2018). Soil-transmitted Helminth infection in the Tiko Health District, South West Region of Cameroon: A post-intervention survey on prevalence and intensity of infection among primary school children. Pan Afr. Med. J..

[17] Palmeirim M.S., Hürlimann E., Knopp S., Speich B., Belizario V., Joseph S.A., Vaillant M., Olliaro P., Keiser J. (2018). Efficacy and safety of co-administered ivermectin plus albendazole for treating soil-transmitted helminths: A systematic review, meta-analysis and individual patient data analysis. PLoS Neglected Trop. Dis..

[18] Gebrezgabiher G., Yewhalaw D., Ayana M., Hailu A., Mekonnen Z. (2022). Impact of ivermectin mass drug administration on burden of soil-transmitted helminths in onchocerciasis control and elimination programs, Yeki district, southwest Ethiopia. PLoS ONE.

[19] de Vos A.S., Stolk W.A., Coffeng L.E., de Vlas S.J. (2021). The impact of mass drug administration expansion to low onchocerciasis prevalence settings in case of connected villages. PLoS Neglected Trop. Dis..

[20] Metcalfe C. (1999). Biostatistics: A Foundation for Analysis in the Health Sciences.

[21] Mbondoukwe P., Mboumba P., Mondouo F., Kombila M., Akotet M. (2016). Prevalence of Soil-transmitted Helminths and Intestinal Protozoa in Shanty Towns of Libreville, Gabon. Int. J. Trop. Dis. Health.

[22] Kato K.M.M. (1954). Comparative examinations. Jpn. J. Parasitol..

[23] Harada Y., Mori O.J.Y.A.M. (1955). A New Method for culturing Hook Worm. Yonago Acta Med..

[24] Ash L.R., Orihe T.C., LSavioli L. (2019). Bench Aids for the Diagnosis of Intestinal Parasites.

[25] Vlaminck J., Fischer P.U., Weil G.J. (2015). Diagnostic Tools for Onchocerciasis Elimination Programs. Trends Parasitol..

[26] Hotterbeekx A., Perneel J., Mandro M., Abhafule G., Siewe Fodjo J.N., Dusabimana A., Abrams S., Kumar-Singh S., Colebunders R. (2020). Comparison of Diagnostic Tests for Onchocerca volvulus in the Democratic Republic of Congo. Pathogens.

[27] Engels D., Zhou X.N. (2020). Neglected tropical diseases: An effective global response to local poverty-related disease priorities. Infect Dis. Poverty.

[28] Duerr H.P., Eichner M. (2010). Epidemiology and control of onchocerciasis: The threshold biting rate of savannah onchocerciasis in Africa. Int. J. Parasitol..

[29] Eyang-Assengone E.R., Makouloutou-Nzassi P., Mbou-Boutambe C., Bangueboussa F., Atsame J., Boundenga L. (2023). Status of Onchocerciasis Elimination in Gabon and Challenges: A Systematic Review. Microorganisms.

[30] Mintsa Nguema R., Mavoungou J.F., Mengue Me Ngou-Milama K., Mabicka Mamfoumbi M., Koumba A.A., Sani Lamine M., Diarra A., Nkone Asseko G., Mourou J.R., Bouyou Akotet M.K. (2018). Baseline Mapping of Schistosomiasis and Soil Transmitted Helminthiasis in the Northern and Eastern Health Regions of Gabon, Central Africa: Recommendations for Preventive Chemotherapy. Trop. Med. Infect. Dis..

[31] Hassen M., Mohammed A., Endeshaw T., Seid T., Samuel F., Asmare T., Birhanu H., Bekele F., Yayeh A., Seife F. (2023). Integrated Prevalence Assessment of Wuchereria bancrofti and Onchocerca volvulus in Three Co-Endemic Districts of Gambella Region, Ethiopia. Am. J. Trop. Med. Hyg..

[32] Moutongo Mouandza R., Mourou J.R., Moutombi Ditombi B., Roger Sibi Matotou H., Ekomi B., Bouyou-Akotet M.K., Mawili-Mboumba D.P. (2023). Sociodemographics, Clinical Factors, and Biological Factors Associated with Loiasis in Endemic Onchocerciasis Areas in Southern Gabon. Am. J. Trop. Med. Hyg..

[33] Tekalign E., Bajiro M., Ayana M., Tiruneh A., Belay T. (2019). Prevalence and Intensity of Soil-Transmitted Helminth Infection among Rural Community of Southwest Ethiopia: A Community-Based Study. Biomed. Res. Int..

[34] Dieye Y., Storey H.L., Barrett K.L., Gerth-Guyette E., Di Giorgio L., Golden A., Faulx D., Kalnoky M., Ndiaye M.K.N., Sy N. (2017). Feasibility of utilizing the SD BIOLINE Onchocerciasis IgG4 rapid test in onchocerciasis surveillance in Senegal. PLoS Neglected Trop. Dis..

[35] WHO (2016). Guidelines for Stopping Mass Drug Administration and Verifying Elimination of Human Onchocerciasis: Criteria and Procedures.

[36] Yang D., Yang Y., Wang Y., Yang Y., Dong S., Chen Y., Zho Y.U. (2018). Prevalence and Risk Factors of Ascaris lumbricoides, Trichuris trichiura and Cryptosporidium Infections in Elementary School Children in Southwestern China: A School-Based Cross-Sectional Study. Int. J. Environ. Res. Public Health.

[37] Evans D.S., Unnasch T.R., Richards F.O. (2015). Onchocerciasis and lymphatic filariasis elimination in Africa: It’s about time. Lancet.

[38] Dolo H., Coulibaly Y.I., Dembele B., Guindo B., Coulibaly S.Y., Dicko I., Doumbia S.S., Dembele M., Traore M.O., Goita S. (2019). Integrated seroprevalence-based assessment of Wuchereria bancrofti and Onchocerca volvulus in two lymphatic filariasis evaluation units of Mali with the SD Bioline Onchocerciasis/LF IgG4 Rapid Test. PLoS Neglected Trop. Dis..

[39] Asfaw M.A., Gezmu T., Wegayehu T., Bekele A., Hailemariam Z., Masresha N., Gebre T. (2020). Soil-transmitted helminth infections among pre-school aged children in Gamo Gofa zone, Southern Ethiopia: Prevalence, intensity and intervention status. PLoS ONE.

[40] Fleming F.M., Brooker S., Geiger S.M., Caldas I.R., Correa-Oliveira R., Hotez P.J., Bethony J.M. (2006). Synergistic associations between hookworm and other helminth species in a rural community in Brazil. Trop. Med. Int. Health.

[41] Blackwell A.D., Martin M., Kaplan H., Gurven M. (2013). Antagonism between two intestinal parasites in humans: The importance of co-infection for infection risk and recovery dynamics. Proc. Biol. Sci..

[42] Dunn J.C., Turner H.C., Tun A., Anderson R.M. (2016). Epidemiological surveys of, and research on, soil-transmitted helminths in Southeast Asia: A systematic review. Parasites Vectors.

[43] Halwindi H., Magnussen P., Olsen A., Lisulo M. (2017). Potential Contribution of Adult Populations to the Maintenance of Schistosomiasis and Soil-Transmitted Helminth Infections in the Siavonga and Mazabuka Districts of Zambia. J. Biosoc. Sci..

[44] Faulkner H., Turner J., Behnke J., Kamgno J., Rowlinson M.C., Bradley J.E., Boussinesq M. (2005). Associations between filarial and gastrointestinal nematodes. Trans. R. Soc. Trop. Med. Hyg..

[45] Gebrezgabiher G., Mekonnen Z., Yewhalaw D., Hailu A. (2019). Reaching the last mile: Main challenges relating to and recommendations to accelerate onchocerciasis elimination in Africa. Infect. Dis. Poverty.

[46] Speich B., Ali S.M., Ame S.M., Bogoch I.I., Alles R., Huwyler J., Albonico M., Hattendorf J., Utzinger J., Keiser J. (2015). Efficacy and safety of albendazole plus ivermectin, albendazole plus mebendazole, albendazole plus oxantel pamoate, and mebendazole alone against Trichuris trichiura and concomitant soil-transmitted helminth infections: A four-arm, randomised controlled trial. Lancet Infect. Dis..

[47] Le B., Clarke N.E., Hii S.F., Byrne A., Khattak A., Lake S., Lazu E., Wickham S., Wand H., Olsen N. (2024). Effectiveness of one and two doses of ivermectin mass drug administration in reducing the prevalence and intensity of soil-transmitted helminth (STH) infections in Western Province, Solomon Islands: A cluster-randomised, before-after analysis. Lancet Reg. Health West. Pac..

[48] Clarke N.E., Doi S.A.R., Wangdi K., Chen Y., Clements A.C.A., Nery S.V. (2019). Efficacy of Anthelminthic Drugs and Drug Combinations Against Soil-transmitted Helminths: A Systematic Review and Network Meta-analysis. Clin. Infect. Dis. Off. Publ. Infect. Dis. Soc. Am..

[49] Belizario V.Y., Amarillo M.E., de Leon W.U., de los Reyes A.E., Bugayong M.G., Macatangay B.J. (2003). A comparison of the efficacy of single doses of albendazole, ivermectin, and diethylcarbamazine alone or in combinations against Ascaris and *Trichuris* spp.. Bull. World Health Organ..

